# Socioeconomic inequalities in modern contraceptive use among women in Benin: a decomposition analysis

**DOI:** 10.1186/s12905-023-02601-y

**Published:** 2023-08-23

**Authors:** Eugene Budu, Louis Kobina Dadzie, Tarif Salihu, Bright Opoku Ahinkorah, Edward Kwabena Ameyaw, Richard Gyan Aboagye, Abdul-Aziz Seidu, Sanni Yaya

**Affiliations:** 1 Korle Bu Teaching Hospital, P. O. Box, 77, Accra, Ghana; 2 Department of Population and Health, University of Cape Coast, Cape Coast, Ghana; 3 Cape Coast Teaching Hospital, Cape Coast, Ghana; 4 School of Public Health, Faculty of Health, University of Technology Sydney, Sydney, Australia; 5 REMS Consult Limited, Sekondi-Takoradi, Western Region Ghana; 6 Institute of Policy Studies and School of Graduate Studies, Lingnan University, Tuen Mun, Hong Kong; 7 L & E Research Consult Ltd, Upper West Region, Wa, Ghana; 8 Department of Family and Community Health, Fred N. Binka School of Public Health, University of Health and Allied Sciences, Ho, Ghana; 9 College of Public Health, Medical and Veterinary Sciences, James Cook University, Townsville, Australia; 10 Centre For Gender and Advocacy, Takoradi Technical University, P.O.Box 256, Takoradi, Ghana; 11 School of International Development and Global Studies, University of Ottawa, Ottawa, K1N 6N5 Canada; 12 The George Institute for Global Health, Imperial College London, London, UK

**Keywords:** Modern contraceptive, Socioeconomic, Inequality, Benin, Demographic and Health Survey

## Abstract

**Background:**

Contraceptive use is crucial to achieving Sustainable Development Goal 3. Evidence of socioeconomic inequality in the use of modern contraceptives is essential to address the developing inequality in its utilisation given the low prevalence of contraceptive use among women in Benin. This study examined the socioeconomic inequalities in modern contraceptive use among women in Benin.

**Methods:**

We performed a cross-sectional analysis of the 2017-18 Benin Demographic and Health Survey data. A weighted sample of 7,360 sexually active women of reproductive age was included in the study. We used a concentration curve to plot the cumulative proportion of women using modern contraception. Decomposition analysis was conducted to determine factors accounting for the socioeconomic disparities in modern contraceptive use.

**Results:**

We noted that the richest women had higher odds of modern contraceptive use (adjusted odds ratio [aOR] = 1.67, CI = 1.22–2.30) compared to the poorest women. Other factors that showed significant associations with modern contraception use were age, marital status, religious affiliation, employment status, parity, women’s educational level, and ethnicity. We found that modern contraceptive use is highly concentrated among the rich, with rich women having a higher propensity of using modern contraception relative to the poor. Also, the disadvantaged to modern contraceptive use included the poor, those aged 45–49, married women, those working, those with four or more live births, rural residents, and women of Bariba and related ethnicity. Conversely, favourable concentration in modern contraceptive use was found among the rich, women aged 20–24, the divorced, women with two live births, the highly educated, those with media exposure, and women of Yoruba and related ethnicity.

**Conclusion:**

The study has shown that wealthy women are more likely to utilize contraceptives than the poor. This is because wealthy women could afford both the service itself and the travel costs to the health facility, hence overcoming any economic barriers to using modern contraception. Other factors such as age, marital status, religion, employment status, parity, mother’s educational level, and ethnicity were associated with contraceptive use in Benin. The Benin government and other stakeholders should develop family planning intercession techniques that address both the supply and demand sides of the equation, with a focus on reaching the illiterate and under-resourced population without admittance to modern contraception.

## Background

One of the most economical methods for advancing reproductive health and boosting socioeconomic growth worldwide is the use of modern contraceptives, which have received widespread acceptance [1, 2]. As echoed in Sustainable Development Goals (SDG) 3 and 5, the SDGs place a special emphasis on the requirement of guaranteeing that everyone has access to sexual and reproductive health services and rights by the year 2030 [3, 4]. The use of modern contraceptives is essential to achieving SDG 3, target 3.7, which accentuates that by 2030, women should have widespread access to sexual and reproductive healthcare services globally [5, 6].

Approximately 308 million unwanted births were avoided in 2017 through the use of modern contraceptives according to a report [7]. Additionally, 67 million unwanted pregnancies might be prevented if the demand for modern contraception was satisfied [6, 7]. According to Hubacher and Trussell (2015), a modern contraceptive method is a drug or medical techinque that prevents sexual activity from leading to pregnancy. Modern contraception could reduce maternal mortalities from 308,000 to 84,000 and newborn deaths from 2.7 million to 538,000 each year if it is made available to women who want to avoid getting pregnant [6, 8]. These decreases in inadvertent pregnancies and maternal and infant deaths can help achieve SDG 3, targets 3.1 and 3.2, which aim to achieve the respective goals of decreasing the worldwide maternal mortality ratio to less than 70 per 100,000 live births and eliminating all avoidable mortalities of children under the age of five by 2030 [6, 7].

Globally, there are roughly 1.9 billion fertile women. Among them, 1.1 billion require family planning [7, 9]. Just 842 million people utilized modern contraceptives in 2019 [7, 9]. Yet, almost 190 million women aged 15–49 who desire to prevent pregnancy did not utilize any kind of contraception globally, up from 156 million in 2000 [6, 10]. In low-and middle-income countries, 214 million women who desired to prevent pregnancy did not use any kind of contraception in 2019 [6, 7]. In sub-Saharan Africa (SSA), most women who desire to prevent pregnancy do not utilize any form of contraceptive method [10]. Evidence from the United Nations Department of Economic and Social Affairs and Population Division showed that 15 sub-Saharan African countries had more than 20% of the world’s unmet family planning needs [6, 10]. Additionally, a survey indicated that there were 51 million females of reproductive age who needed access to modern contraceptives [9].

Internationally, the issue of inequalities in contraceptive use is garnering attention in the field of public health, with a previous study showing that the underprivileged segments of society experience the poorest results from modern contraceptive use [11]. Inequalities in family planning services, such as modern contraception, affect Beninese women, as they do in other sub-Saharan African countries. The Safe Motherhood Initiative, which aims to decrease problems and deaths associated with pregnancy in developing countries, heavily relies on family planning [12, 13]. All women should, by necessity, have access to family planning services, including modern contraceptives, regardless of their social, economic, or geographic circumstances [13]. More specifically, countries like Benin still struggles with significant inequalities in the use of modern contraceptives while having made progress in enhancing family planning services and decreasing maternal morbidity and mortality in general. Inequality in access to safe maternal health services, such as the use of modern contraceptives, and higher morbidity and mortality rates are common among disadvantaged women [13].

Despite the initiatives and pledges to increase the use of modern contraceptives, usage rates in the Benin Republic remain low, even though awareness of contraceptive techniques is ubiquitous throughout the nation (knowledge of any method is at 85%) [14, 15]. In 2018, 12% of women used a modern form of contraception, up from 6% to 2006 [14]. In addition, almost one in five (or 48%) adolescent girls between the ages of 15 and 19 who engage in sexual activity are either pregnant or have given birth to a child previously[14, 16]. According to the country’s authorities, just 5.4% of women aged 15 to 24 in Benin used modern methods of contraception in 2017 [16]. Current statistics reveal that the overall fertility rate for all women between the ages of 15 and 49 who are fertile is 5.7, while the prevalence of modern contraception is believed to be 12% [14, 16]. Studies conducted by Chae et al. [17] and Ahissou et al.[16] have shown that 19% of all pregnancies in the country were unplanned. In 2017, the maternal mortality ratio and newborn mortality rate continued to be lofty at 397 per 100,000 live births and 30 per 1,000 live births, respectively [16, 18].

In Benin, there are considerable differences in how often women utilise modern contraceptives as a result of social, economic, and geographic reasons [13]. The socioeconomic inequalities between the various social classes have become an important issue in the use of modern contraceptives in Benin [13]. Evidence on socioeconomic inequalities in the use of modern contraceptives is crucial in light of the SDGs to address the growing inequality in family planning services including modern contraceptives use in Benin. Such proof would encourage a more reasonable delivery of modern contraceptive methods and more effective use of limited resources in Benin [19]. This will eventually ensure equitable access to modern contraceptives among Beninese women. Using data from the Benin Demographic and Health Survey (BDHS), we examined the socioeconomic inequalities in modern contraceptive use among women in Benin.

## Methods

### Data source and study design

Data for this study was obtained from the 2017–2018 BDHS. Specifically, we used data from the individual recode (IR) file of the BDHS. BDHS is part of several surveys obtained from the Monitoring and Evaluation to Assess and Use Results Demographic and Health Surveys (MEASURE DHS) Program, which contain information on some issues on population, health and nutrition including contraception use. The DHS is a comparatively nationally representative survey conducted in over 85 low-and-middle-income countries worldwide [20]. DHS employed a descriptive cross-sectional design. The survey adopted a two-stage sampling design. The first stage was characterised by the selection of clusters across urban and rural locations from the entire nation. These made up enumeration areas for the study. The second stage involved the selection of households from the predefined clusters. Details of the methodologies employed in the various rounds of the surveys can be found in the final reports [14]. Standardized structured questionnaires were used to collect data from the respondents on health indicators including contraceptive use. We included 7,360 sexually active women of reproductive age who had complete observations on variables of interest in the final analysis. The dataset used is free and available at https://dhsprogram.com/data/available-datasets.cfm. This paper was drafted with reference to the Strengthening the Reporting of Observational Studies in Epidemiology guidelines.

### Variables

#### Outcome variable

Utilisation of modern contraceptive method was the outcome variable in the study which was derived from the question: “Which method type of contraceptive does [NAME] currently use?”. Responses to this question were “no method” =0, “folkloric method” =1 “traditional method” =2 and “modern method”=3. For this study, women who responded no method, folkloric, and traditional method were categorised as “0” which represented “other methods” while the modern method was labelled “1” [6, 21–24].

#### Independent variable

Household wealth index was considered the main explanatory variable. The wealth index was calculated by the BDHS using several indicator variables. These indicators included elements about the household’s possessions, traits, ownerships, and utility services, including floor and roof materials, the presence of technological items like televisions, cell phones, and refrigerators, the availability of a source of drinking water for cooking and washing, the type of cooking fuel used, restrooms, and land and livestock ownership, among other things. After standardizing the variables, principal component analysis was performed on these indicator variables, and the wealth index was constructed using the determined factor loadings. Afterwards, the wealth index was sorted and divided into five portions, each of 20%. We used the categories of wealth index that were generated by the DHS as follows: poorest, poorer, middle, richer, and richest in the BDHS dataset.

#### Covariates

Nine other variables were considered for this study. These variables included age (15–19, 20–24, 25–29, 30–34, 35–39, 40–44, and 45–49), marital status (not married and married), religion (Christianity, Islam, and other), employment status (not working and working), number of live births (no birth, one birth, two births, three births, and four or more births), exposure to mass media (no and yes), educational level (no education, primary, and secondary/higher), place of residence (urban and rural), and ethnicity (Adja and related, Bariba and related, Dendi and related, Fon and related, Yoa, loka and related, Betamaribe and related, Peulh and related, and Yoruba and related). The explanatory variables considered in this study were selected based on their association with modern contraceptive use [1, 2, 13, 16].

### Statistical analyses

Missing observations on variables of interest were dropped during the analysis. The prevalence of modern contraceptive use by sociodemographic factors was determined using univariate and bivariate analysis (Table 1). We used binary. logistic regression analysis to examine the factors associated with the utilisation of modern contraceptives. Model I and II were the bivariable and multivariable binary regression analyses, respectively. The results in Model I were presented as crude odds ratio (cOR) with their respective 95% confidence interval (CI), whilst the results in Model II were presented as adjusted odds ratio (aOR) with their 95% CI (Table 2).

**Table 1:** Prevalence of modern contraceptive use by socio-demographic characteristics

					Modern contraceptive use (%)
Variables	Weighted sample (N)	Weighted percentage (%)	Unweighted N	Unweighted %	
**Women’s age**					
15–19	716	9.7	709	9.6	13.6
20–24	1,432	19.5	1418	19.3	14.9
25–29	1,617	22.0	1613	21.9	16.6
30–34	1,287	17.5	1277	17.4	20.7
35–39	1,058	14.4	1083	14.7	18.2
40–44	718	9.7	722	9.8	17.9
45–49	530	7.2	538	7.3	12.9
**Marital status**					
Not married	2267	30.8	2258	30.7	19.1
Married	5,093	69.2	5102	69.3	15.7
**Religious affiliation**					
Christianity	4,037	54.9	4079	55.4	18.5
Islam	2,274	30.9	2213	30.1	15.2
Others	1049	14.2	1068	14.5	13.5
**Employment status**					
Not working	1,153	15.7	1149	15.6	13.4
Working	6,207	84.3	6211	84.4	17.4
**Number of live births**					
Zero	1,048	14.2	1063	14.4	12.6
One	1,024	13.9	1013	13.8	14.8
Two	1,108	15.1	1111	15.1	13.5
Three	1,066	14.5	1062	14.4	15.1
Four or more	3,114	42.3	3111	42.3	20.6
**Women’s educational level**					
No education	4,524	61.5	4495	61.1	14.5
Primary	1,377	14.7	1355	18.4	18.4
Secondary/Higher	1459	19.8	1510	20.5	22.3
**Exposure to mass media**					
Not exposed	4,814	65.4	4848	65.9	16.1
Exposed	2,546	34.6	2512	34.1	18.0
**Place of residence**					
Urban	2,966	40.3	3105	42.2	19.7
Rural	4,394	59.7	4255	57.8	14.8
**Wealth index**					
Poorest	1,314	17.8	1355	18.4	13.7
Poorer	1,383	18.8	1373	18.6	13.8
Middle	1,476	20.1	1405	19.1	15.2
Richer	1,595	21.7	1539	20.9	17.1
Richest	1,591	21.6	1688	22.9	23.0
**Ethnicity**					
Adja and related	871	11.8	888	12.1	15.9
Bariba and related	1,021	13.9	955	13.0	20.9
Dendi and related	490	6.7	463	6.3	17.4
Fon and related	2,788	37.9	2754	37.4	18.3
Yoa, loka and related	244	3.3	241	3.3	8.4
Betamaribe and related	424	5.8	455	6.2	16.7
Peulh and related	649	8.8	672	9.1	10.4
Yoruba and related	873	11.8	932	12.7	10.8

* *p*<0.05, ** *p*<0.01, *** *p*<0.001

**Table 2:** Binary Logistic Regression of sociodemographic characteristics and utilisation of modern contraceptives

Variables	Model I (cOR)	Model II (aOR)
Wealth index		
Poorest	Reference (1.0)	Reference (1.0)
Poorer	1.02 (0.77–1.33)	0.98 (0.74–1.31)
Middle	1.13 (0.87–1.48)	1.08 (0.81–1.46)
Richer	1.31 (1.00-1.71)	1.18 (0.88–1.59)
Richest	1.89^***^ (1.47–2.43)	1.71^***^ (1.24–2.34)
**Women’s age**		
15–19	Reference (1.0)	Reference (1.0)
20–24	1.12 (0.84–1.48)	0.91 (0.66–1.25)
25–29	1.27 (0.97–1.66)	0.69^*^ (0.49–0.98)
30–34	1.67^***^ (1.28–2.18)	0.69^*^ (0.48–0.98)
35–39	1.42^*^ (1.06–1.90)	0.52^***^ (0.35–0.77)
40–44	1.39^*^ (1.03–1.88)	0.52^***^ (0.34–0.78)
45–49	0.95 (0.65–1.38)	0.33^***^ (0.20–0.53)
**Marital status**		
Not married	Reference (1.0)	Reference (1.0)
Married	0.79^***^ (0.68–0.92)	0.72^***^ (0.61–0.85)
**Religious affiliation**		
Christianity	Reference (1.0)	Reference (1.0)
Islam	0.79^*^ (0.65–0.96)	0.80 (0.61–1.06)
Others	0.69^***^ (0.56–0.85)	0.76^*^ (0.60–0.95)
**Employment status**		
Not working	Reference (1.0)	Reference (1.0)
Working	1.37^**^ (1.08–1.73)	1.27^*^ (1.01–1.60)
**Number of live births**		
Zero	Reference (1.0)	Reference (1.0)
One	1.21 (0.92–1.57)	1.62^**^ (1.21–2.16)
Two	1.08 (0.82–1.43)	1.67^**^ (1.20–2.34)
Three	1.23 (0.94–1.61)	2.34^***^ (1.69–3.23)
Four or more	1.79^***^ (1.44–2.23)	5.07^***^ (3.63–7.09)
**Women’s educational level**		
No education	Reference (1.0)	Reference (1.0)
Primary	1.33^***^ (1.11–1.59)	1.39^***^ (1.14–1.68)
Secondary/Higher	1.70^***^ (1.45–1.99)	1.95^***^ (1.60–2.38)
**Exposure to mass media**		
Not exposed	Reference (1.0)	Reference (1.0)
Exposed	1.14 (0.98–1.33)	1.07 (0.90–1.26)
**Place of residence**		
Urban	Reference (1.0)	Reference (1.0)
Rural	0.71^***^ (0.60–0.84)	0.90 (0.73–1.09)
**Ethnicity**		
Adja and related	Reference (1.0)	Reference (1.0)
Bariba and related	1.40^*^ (1.04–1.88)	1.94^***^ (1.36–2.78)
Dendi and related	1.11 (0.73–1.69)	1.58 (0.95–2.62)
Fon and related	1.18 (0.93–1.50)	1.06 (0.83–1.36)
Yoa, loka and related	0.49^*^ (0.25–0.96)	0.71 (0.34–1.46)
Betamaribe and related	1.06 (0.71–1.58)	1.42 (0.96–2.10)
Peulh and related	0.61^*^ (0.42–0.90)	0.96 (0.61–1.52)
Yoruba and related	0.90 (0.66–1.23)	0.96 (0.69–1.33)

* p < 0.05, ** p < 0.01, *** p < 0.001

### Inequality measurement

We created a concentration curve that plotted the cumulative proportion of respondents using modern contraception against the cumulative proportion of respondents reporting wealth status to assess socioeconomic disparities in women’s usage of modern contraception. The concentration index was then calculated using a formula created and employed by Kakwani [25] and Jenkins [26]. The model estimation is described as;

$$CIX=\frac{2}{\mu }cov\left(h, r\right);$$

Where;

CIX = the concentration index.

$\mu$= the weighted mean of the indicator whose concentration is to be calculated (use of modern contraception)

$$cov$$= covariance

$h$= variable whose concentration is to be calculated

$r$= fractional rank of people in the distribution of variable by which concentration will be calculated (household wealth)

$cov\left(h, r\right)$= covariance between h and r

The index’s values range from − 1 to + 1, with values closer to + 1 indicating stronger concentration in the variable’s upper quintile and vice versa.

#### Decomposition of CIX

Following the creation of dummy variables for the independent variables, a decomposition analysis was conducted to determine the various contributions made by the independent factors that account for the inequality. This was fitted into the regression model developed by O’Donnell et al. [27] as follows;

$$y = {a_0} + \sum\limits_k {\beta kXk + \varepsilon }$$

Where.

$\beta k$= coefficient of kth explanatory variable

$Xk$= kth explanatory variable

$\epsilon =$random error term

This equation was utilized for the decomposition of the usage of modern contraception as follows

$$CI = \sum\limits_k {\left( {\frac{{\beta kXk}}{\mu }} \right)Ck + GC/\mu ;}$$

where.

Xk = mean of kth explanatory variable.

Ck = concentration of kth explanatory variable.

$\left(\frac{\beta kXk}{\mu }\right)$= is the elasticity of the use of modern contraception with respect to the kth explanatory variable

$GC/\mu$= aspect of CIX that cannot be explained by the explanatory variables

**Fig. 1:**
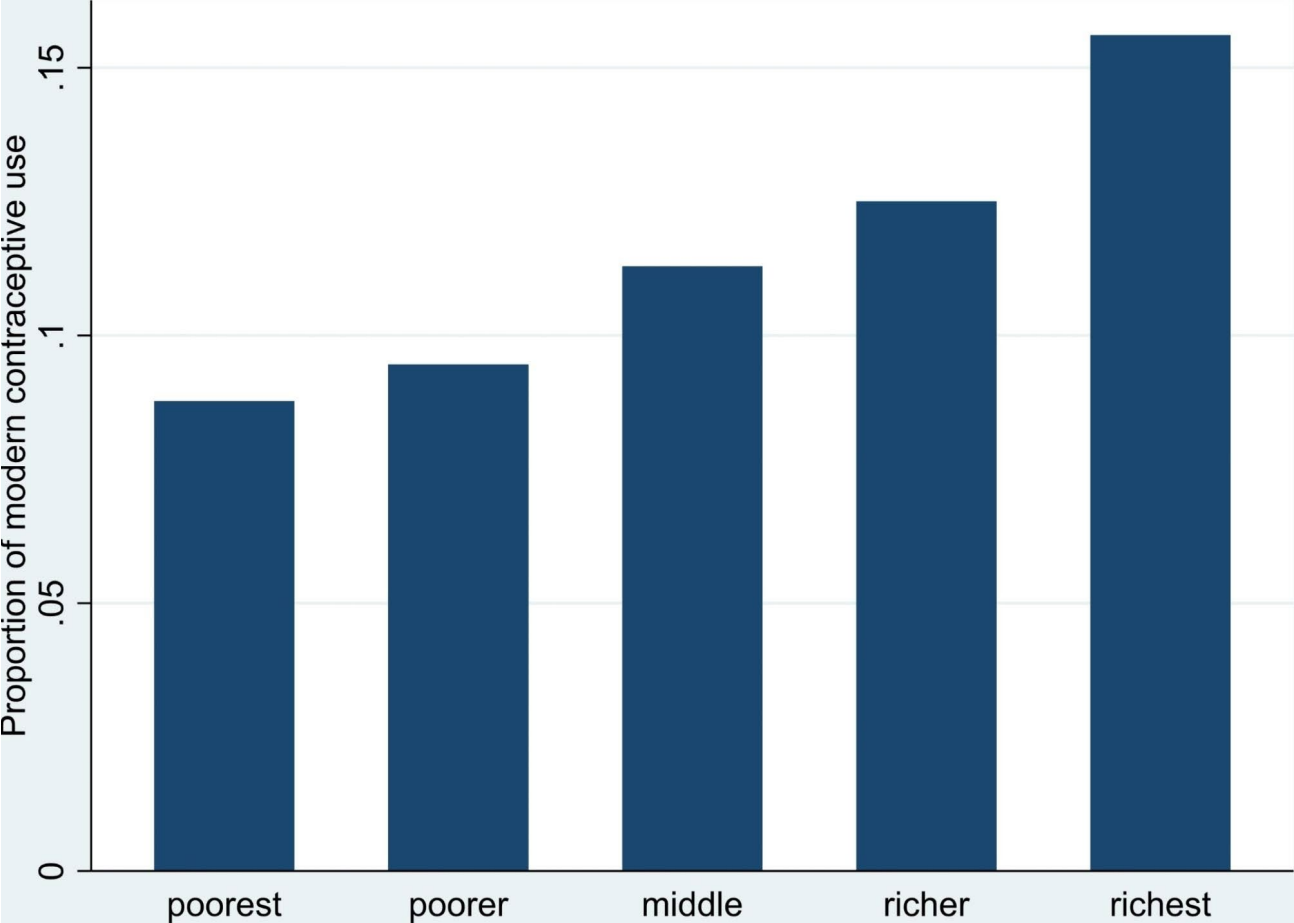
Modern contraceptive use by wealth status

In addition, a graphic depicting the percentage of women who use modern contraceptives according to their wealth status was employed in the study (Fig. 1). Stata 16.0 was used to analyze all the data. Before the analysis, we designated the data as survey data, and the outcomes were weighted to take into account sampling irregularities.

### Ethical approval

The DHS reports that ethical clearances were obtained from the Ethics Committee of ORC Macro Inc. as well as Ethics Boards of partner organisations of the various countries such as the Ministries of Health. The DHS follows the standards for ensuring the protection of respondents’ privacy. ICF International ensures that the survey complies with the U.S. Department of Health and Human Services’ regulations for the respect of human subjects. This was a secondary analysis of data and therefore no further approval was required since the data is available in the public domain. Further information about the DHS data usage and ethical standards is available at http://goo.gl/ny8T6X.

## Results

### Proportion of modern contraceptive use across wealth index

Figure 1 shows that the proportion of modern contraceptive use among women in Benin is highest among those of the richest wealth index and lowest among those of the poorest wealth index.

#### Prevalence of modern contraception use by socio-demographic characteristics

Modern contraceptive use was profound among those in the 30–34 age range (20.7%). A significant proportion of the divorced (22.4%) and Christians (18.5%) reported modern contraceptive use. Modern contraceptive use dominated among those who were working (17.4%). Those who had four or more births recorded the highest prevalence of modern contraceptive use (20.6%). Those with higher education recorded the highest prevalence of modern contraceptive use (31.6%). Modern contraceptive use was common among those with media exposure (18.0%). Modern contraceptive use was also high among urban dwellers (19.7%). The richest women recorded the highest prevalence of modern contraceptive use (23.0%). Regarding ethnicity, 20.9% of women of Bariba and related ethnicity reported modern contraceptive use (Table 1).

#### Logistic regression of socio-demographic characteristics and utilisation of modern contraceptives

Table 2 presents the results of the association between the socio-demographic characteristics and the use of modern contraceptives. The results showed that women in the richest wealth index were more likely to use modern contraceptives (aOR = 1.67, CI = 1.22–2.30) compared to the poorest women. Other factors that showed significant associations with modern contraception use were age, marital status, religious affiliation, employment status, parity, women’s educational level, and ethnicity (Table 2).

#### Inequality in modern contraceptive use by wealth

**Fig. 2:**
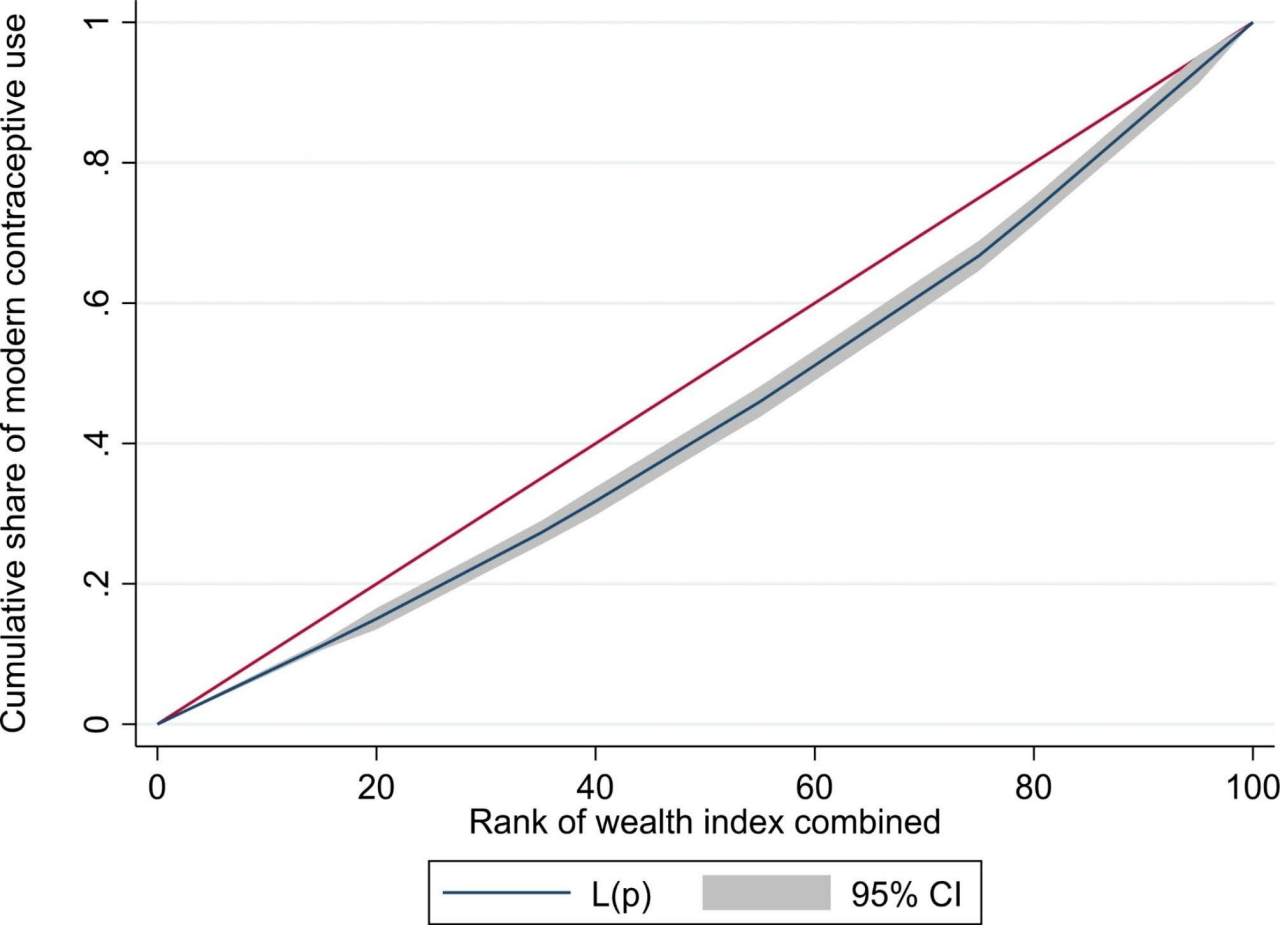
Inequality in modern contraceptive use by wealth index

Figure 2 shows the concentration curve of modern contraceptive use with respect to the wealth index of the women. A 45° red line indicates the line of equality and the black line is the line of concentration curve. Any deviation from the line indicates the presence of inequality. If the concentration curve is below the line of equality it indicates that modern contraceptive use is concentrated among the women with higher wealth status and the concentration curve above the line of equality indicates that that modern contraceptive use is concentrated among the women with lower wealth status. The figure demonstrates inequality in modern contraception use among the women. The red diagonal straight line is the line of equality, whilst the black line beneath the red line is the concentration curve (CC). The farther the CC from the equality line, the higher the concentration of modern contraceptive use among the rich. Figure 2 has shown that modern contraceptive use is highly concentrated among the rich. In other words, the figure depicts that rich women are at an advantage in terms of using modern contraceptive. Hence, there is a pro-rich inequality in modern contraceptive use, as the concentration curve lies below the equality line.

In Table 3, we presented results from the decomposition analysis by illustrating how the socio-demographic characteristics of the women contribute to inequality in modern contraceptive use. The results are presented in absolute (i.e. concentration index) and percentage contribution (i.e. adjusted percentage contribution of inequality) terms. The disadvantaged concerning modern contraception use included the poor, those aged 45–49, married women, those working, those with four or more live births, rural residents, and women of Bariba and related ethnicity. Conversely, favourable concentration in modern contraceptive use was noted among the rich, women aged 20–24, the divorced, women with two live births, the highly educated, those with media exposure, and women of Yoruba and related ethnicity.

**Table 3:** Contribution of socio-demographic characteristics based on the decomposition of concentration index analysis for modern contraceptive use

Variables	Elasticity	Concentration index	Absolute contribution	Percentage contribution
**Wealth index**				
Poorest				
Poorer	-0.0019	-0.4550	0.0008	0.7829
Middle	0.0086	-0.0664	-0.0006	-0.5281
Richer	0.0194	0.3509	0.0068	6.2977
Richest	0.0613	0.7838	0.4806	44.3766
**Women’s age**				
15–19				
20–24	-0.0099	0.0024	-0.0000	-0.0215
25–29	-0.0430	0.0115	-0.0005	-0.4571
30–34	-0.0351	0.0260	-0.0009	-0.8427
35–39	-0.0499	0.0585	-0.0029	-2.6928
40–44	-0.0343	-0.0324	0.0011	1.0263
45–49	-0.0427	-0.0377	00016	1.4849
**Marital status**				
Not married				
Married	-0.1201	-0.0339	0.0041	3.7558
**Religious affiliation**				
Christianity				
Islam	-0.0359	-0.1396	0.0050	4.6279
Others	-0.0213	-0.2111	0.0045	4.1449
**Employment status**				
Not working				
Working	0.1080	0.0089	0.0010	0.8866
**Number of live births**				
Zero				
One	0.0355	0.0614	0.00218	2.0118
Two	0.0412	0.1090	0.0045	4.1453
Three	0.0654	0.0461	0.0030	2.7828
Four or more	0.3654	-0.1038	-0.0379	-35.0064
**Women’s educational level**				
No education				
Primary	0.0325	0.1548	0.0050	4.6463
Secondary/Higher	0.0706	0.4028	0.0284	26.2462
**Exposure to mass media**				
Not exposed				
Exposed	0.0120	0.1075	0.0013	1.1957
**Place of residence**				
Urban				
Rural	-0.0352	0-0.1902	0.0067	6.1802
**Ethnicity**				
Adja and related				
Bariba and related	0.0490	-0.1677	-0.0082	-7.5919
Dendi and related	0.0161	-0.1204	-0.0019	-1.7888
Fon and related	0.0122	0.1945	0.0024	2.1870
Yoa, loka and related	-0.0061	0.0168	-0.0001	-0.0949
Betamaribe and related	0.0107	-0.3353	-0.0036	-3.300
Peulh and related	-0.0019	-0.5509	0.0010	0.9683
Yoruba and related	-0.0029	0.1227	-0.0003	-0.3231

## Discussion

Our study examined the socioeconomic inequalities in women’s usage of modern contraceptives in Benin. Age, marital status, religion, work status, parity, women’s education, wealth index, and ethnicity were revealed to be the most significant socioeconomic determinants of modern contraceptive use in Benin.

Our study revealed disparities in the usage of modern contraceptives among women in Benin. It is well known that the SDGs encourage the lowering of inequalities and provide universal access to health care services, including family planning services. Given the foregoing, in addition to using sexual and reproductive health services, it is also necessary to explore how to reach the most underprivileged group of women about the use of sexual and reproductive health services, including modern contraception, to meet the objectives [3, 13]. The findings of this study showed that the rich have a highly concentrated usage of modern contraceptives. Thus, wealthier women were more likely than poor women to use modern contraception. It illustrates a pro-rich inequality in the use of modern contraceptives and is consistent with the positive concentration index among women who are richer and richest. These findings are consistent with a large body of literature from SSA, which indicates that the impoverished population have unequal access to modern contraceptive methods [28], and are in greater danger of not using sexual and reproductive health services, including contraception [19]. Basically, disadvantaged groups with elevated poverty and low educational levels frequently have greater levels of inequity in modern contraceptive use among women [29].

Women with lower socioeconomic positions use modern contraception less frequently than they should. When seeking sexual and reproductive health care, mothers from more affluent homes are more probable to go to a facility and, if necessary, pay for both the service itself and the travel costs [30, 31]. Additionally, women who had enough income and were employed had a greater chance of utilising modern contraceptives, which aids them to delay childbirth [32]. Our results are consistent with earlier studies which revealed that an improvement in women’s financial standing positively correlates with an upsurge in the use of sexual and reproductive health services, such as the use of modern contraceptives [31, 33]. Medical costs, particularly in Benin’s rural areas, are a substantial barrier to the use of modern contraceptives by women from low-income households. These costs include opportunity costs, transportation expenses, prescription and supply prices, and the price of healthcare services [31]. In several sub-Saharan African countries, including Ghana [34], Malawi [32], Nigeria [35], and Ethiopia [36], wealth has been documented to be the main factor contributing to the disparity in modern contraceptive usage. The reason for this is the economic hardship that using contraception entails. Whereas the wealthiest women may be able to avoid any economic barriers to using modern contraception, impoverished women may not [6].

Additionally, a study conducted in Ghana [37] revealed that inferior socioeconomic features, such as high poverty and low education, were the factors contributing to the inequity in modern contraception among women. This shows that women who are underprivileged and with low levels of education have limited contact with modern contraception. Furthermore, the supply of family planning services, especially modern contraception to the most disadvantaged segments, is severely hampered by the inadequate accessibility of reproductive health services, competent health professionals, and medicines in many African countries [28]. Even though a number of health reforms that have been put in place over the past few years, such as augmented investment in health infrastructure and workforce expansion and retention, improvement in family planning services delivery, the wealth gap found in this study calls for energies to be focused on expanding admittance to education and income-making programs, especially for women from deprived backgrounds [13, 28].

The present study also found rural-urban inequality in contraceptive use among Beninese women. Comparing rural and urban women in Benin, we found that the former had lower probability of using modern contraceptives. The fact that poor rural women frequently have to travel long distances to reach medical facilities and may not be able to afford the transportation expenses could have accounted for their lower likelihood of using modern contraceptives [28]. Our results are consistent with a study from Ghana, where rural residency considerably influenced the observed gap in trained birth attendants between 1998 and 2014 [38]. In Mozambique, earlier research has revealed geographical disparities in resource distribution, with the affluent region receiving much more financial support, including superior social services like health care and reproductive health services [28]. It may be possible to lessen the perceived disparity in contraceptive use in Benin by improving the social and financial re-distribution of these assets within the country [28].

The study also showed that the disparity in contraceptive use among women in Benin was highly influenced by marital status. Unmarried women in our study were more probable than their counterparts to utilize contraceptives, particularly modern techniques. Contraceptive use seems to be the best choice for sexually active women in urban Benin when there is a worry about the stigma of childbirth while studying, which is a common act in Benin [15]. More so, using modern contraception is a viable option for urban single women who wish to continue their education in the context of today’s evolving society, where sexual orientation is becoming an increasingly significant factor. But this result runs counter to that of Abekah-Nkrumah [38], who found that unmarried women were less probable to obtain modern contraception. However, the conclusions of this study are in direct opposition to that of Abekah-Nkrumah [38], which found that unmarried women had a lower likelihood of using modern contraception.

### Strengths and limitations

This study’s key strength is the decomposition of modern contraception inequality using concentration index. Another strength is the use of nationally representative data gathered using a standardized methodologies DHS employed a cross-sectional design and this limits the study’s ability to draw causal inferences. Additionally, modern contraceptive use was assessed using self-reports, hence, there could be a possibility of recall and social desirability biases.

## Conclusions and policy implications

The study has shown that wealthy women are more likely to utilize contraceptives than the poor. Other factors such as age, marital status, religion, employment status, parity, women’s educational level, and ethnicity were associated with contraceptive use in Benin. This study results indicated consistent inequalities in modern contraceptive use in favour of married women, educated, and wealthy women. To eliminate inequality in contraceptive use in Benin, policy-makers need to develop measures for the underserved women (i.e. those with low socioeconomic levels) by eliminating or subsidising expenses which is considered as a core element of an equitable system of user fees for family planning services including modern contraceptive use. Although the Benin government has made significant strides in increasing FP access, including modern contraception, more focus is required on user fee policies for modern contraceptive use. This is especially important to ensure that the poorest, unmarried, and women without formal education receive affordable services and to account for sociodemographic inequality. It also ensures that efforts to compensate facilities for lost user fee revenue are done at the proper levels. Again, as we work toward universal health coverage, policymakers and other key stakeholders should reinforce and deepen mass education programs and policies to increase modern contraceptive usage by facilitating easy access and to address disparities in women’s modern contraceptive use. To enhance women’s knowledge and use of modern contraceptives, such education programs and policies should target poor, single, and uneducated women in rural communities in Benin. This will help Benin accomplish its maternal and child mortality-related SDGs and women’s empowerment. Furthermore, the government of Benin and other interested parties should also develop family planning intercession techniques that address both the supply and demand sides of the issue, with a focus on reaching the illiterate and under-resourced population without admittance to modern contraception.

## Data Availability

Data for this study were sourced from Demographic and Health surveys (DHS) and available here: http://dhsprogram.com/data/available-datasets.cfm.
