# Correction to: Socioeconomic inequalities in modern contraceptive use among women in Benin: a decomposition analysis

**DOI:** 10.1186/s12905-023-02654-z

**Published:** 2023-09-19

**Authors:** Eugene Budu, Louis Kobina Dadzie, Tarif Salihu, Bright Opoku Ahinkorah, Edward Kwabena Ameyaw, Richard Gyan Aboagye, Abdul-Aziz Seidu, Sanni Yaya

**Affiliations:** 1 Korle Bu Teaching Hospital, Accra, P. O. Box, 77, Ghana; 2 Department of Population and Health, University of Cape Coast, Cape Coast, Ghana; 3 Cape Coast Teaching Hospital, Cape Coast, Ghana; 4 School of Public Health, Faculty of Health, University of Technology Sydney, Sydney, Australia; 5 REMS Consult Limited, Sekondi-Takoradi, Western Region, Ghana; 6 Institute of Policy Studies and School of Graduate Studies, Lingnan University, Tuen Mun, Hong Kong; 7 L & E Research Consult Ltd, Upper West Region, Wa, Ghana; 8 Department of Family and Community Health, Fred N. Binka School of Public Health, University of Health and Allied Sciences, Ho, Ghana; 9 College of Public Health, Medical and Veterinary Sciences, James Cook University, Townsville, Australia; 10 Centre For Gender and Advocacy, Takoradi Technical University, Takoradi, P.O. Box 256, Ghana; 11 School of International Development and Global Studies, University of Ottawa, Ottawa, K1N 6N5 Canada; 12 The George Institute for Global Health, Imperial College London, London, UK

Following publication of the original article [1], in this article the reference [27] has been updated as shown below:

O’Donnell O, van Doorslaer E, Wagstaff A, Lindelow M. Analyzing health equity using household survey data [Internet]. Analyzing health equity using household survey data. Washington, DC: World Bank Group; 2007. Available from: http://documents.worldbank.org/curated/en/633931468139502235/Analyzing-health-equity-using-household-survey-data-a-guide-to-tech­niques-and-their-implementation

The original article has been corrected.
